# A Factor Graph Approach to Automated GO Annotation

**DOI:** 10.1371/journal.pone.0146986

**Published:** 2016-01-15

**Authors:** Flavio E. Spetale, Elizabeth Tapia, Flavia Krsticevic, Fernando Roda, Pilar Bulacio

**Affiliations:** 1 CIFASIS-Conicet Institute, Rosario, Argentina; 2 Facultad de Cs. Exactas, Ingeniería y Agrimensura, National University of Rosario, Rosario, Argentina; 3 Facultad Regional San Nicolás, National Technological University, San Nicolás, Argentina; Semmelweis University, HUNGARY

## Abstract

As volume of genomic data grows, computational methods become essential for providing a first glimpse onto gene annotations. Automated Gene Ontology (GO) annotation methods based on hierarchical ensemble classification techniques are particularly interesting when interpretability of annotation results is a main concern. In these methods, raw GO-term predictions computed by base binary classifiers are leveraged by checking the consistency of predefined GO relationships. Both formal leveraging strategies, with main focus on annotation precision, and heuristic alternatives, with main focus on scalability issues, have been described in literature. In this contribution, a factor graph approach to the hierarchical ensemble formulation of the automated GO annotation problem is presented. In this formal framework, a core factor graph is first built based on the GO structure and then enriched to take into account the noisy nature of GO-term predictions. Hence, starting from raw GO-term predictions, an iterative message passing algorithm between nodes of the factor graph is used to compute marginal probabilities of target GO-terms. Evaluations on *Saccharomyces cerevisiae*, *Arabidopsis thaliana* and *Drosophila melanogaster* protein sequences from the GO Molecular Function domain showed significant improvements over competing approaches, even when protein sequences were naively characterized by their physicochemical and secondary structure properties or when loose noisy annotation datasets were considered. Based on these promising results and using *Arabidopsis thaliana* annotation data, we extend our approach to the identification of most promising molecular function annotations for a set of proteins of unknown function in *Solanum lycopersicum*.

## Introduction

A fundamental step, and significant bottleneck, in the acquisition of biological knowledge from genomic data is the characterization of gene products properties. The Gene Ontology (GO) Consortium provides an ontology of terms for describing gene products properties and their relationships in a species-independent manner. The automated association between ontologies and genes and gene products, i.e., the automated annotation of genes, is one of the great challenges to bioinformatics research. In this respect, it is worth mentioning that near 28% of the genes in a model organism like *D. melanogaster* lack of a GO annotation [1]. This percentage grows to 40% in *A. thaliana* [2] and can rise up to 50%, or even higher levels, in non-model organisms like *Helianthus annuus L.* [3]. Standard methods for automated protein-coding gene annotation commonly rely on sequence similarity [4–6] or protein signature [7, 8] searches. However, in absence of clear sequence similarities or definite protein signatures, alternative computational methods for automated gene annotation must be considered. In the case of protein function prediction, one possibility is to use high throughput biological experiments for the identification of protein interaction networks and the prediction of protein functions from those of their interacting partners [9, 10]. However, prediction accuracy in these methods may be limited in the presence of unreliable interactions or lack of sufficient experimentally verified annotation data. Although data integration strategies may reduce these difficulties to some extent, they are only applicable to well-characterized model organisms [11, 12]. Alternatively, these difficulties may be circumvented by using hierarchical ensemble classification techniques [13–16]. By means of these techniques, the problem of automated gene annotation can be cast to that of predicting individual GO-terms within a True Path Graph (TPG) [17], a special kind of Directed Acyclic Graph (DAG) defining the meaning of GO-terms by multiple inheritance. Since predictions of individual GO-terms are expected to be noisy and inconsistent with the TPG, several strategies aiming to leverage them have been proposed in literature.

In [18], a core Bayes net of latent nodes modeling binary-valued GO-term variables was first built using all parent-child relationships established in a predefined GO domain. The core Bayes net was enriched through the addition of leaf nodes modeling real-valued GO-term predictions constrained to follow independent Gaussian distributions over positive and negative latent GO-terms. In practice, leaf nodes are first instantiated by bootstrapped hard-margin SVM classifiers with unthresholded outputs. Afterwards, they are leveraged by a global error-correction strategy based on the computation of a posteriori probabilities of latent GO-terms with standard algorithms for probabilistic inference in Bayesian networks. Although the Bayes network approach was found to be highly effective with a yeast annotation problem involving a GO sub-hierarchy of 105 GO-terms, it could not be used with a mouse annotation problem involving thousands of GO-terms [19]. The main problem is that except for polytree-shaped Bayesian networks, even approximate probabilistic inference in general Bayesian networks is NP-hard [20], i.e., both time and space complexity are exponential in the size of the network in the worst case. To address these scalability issues, a semi-global Bayes error correction approach was considered in [19]. Specifically, multiple polytree-shaped Bayes nets for which linear-time inference algorithms exist were built for each latent GO-term. In presence of rather modest amounts of annotation data, substantial improvements in the precision and recall of raw GO-term predictions were observed. Note, however, that latent GO-term estimations across different sub-Bayes networks may still remain inconsistent.

To overcome the shortcomings of the semi-global Bayes error-correction approach, a heuristic algorithm called True Path Rule (TPR) was proposed in [21]. Originally developed for hierarchical tree-structured ontologies like FunCat [22], the TPR algorithm focuses on the global satisfiability of TPG constraint, i.e., the pathway from a child term to its top-level parent(s) must always be true [23]. In practice, the TPR algorithm performs a bottom-up flow of information that enhances the probability that a class prediction is positive by computing a consensus probability over direct positive descendant classes. This operation may mute a class prediction from positive to negative and in such a case, all descendant classes predicted as positive are muted to negative. In [21], FunCat class predictions were obtained from SVM classifiers with different types of kernels depending on the specific characterization of the input annotation data, e.g., presence/absence of protein domains or protein-protein interaction data. Since probabilistic class predictions were required, sigmoid fitting over SVM outputs was performed [24]. Recently, a revised version of the TPR heuristic valid for DAG structured ontologies like GO has been presented [25]. In this new version, a two-way flow of information is performed. Experimental results on a human annotation problem involving thousands of classes from the human phenotype ontology [26] suggest that the TPR algorithm is indeed effective for improving raw class predictions so they can consistently match a predefined target ontology. It should be noted, however, that consistent TPR class predictions may not be unique and may not be optimal with respect to the minimization of the probability of erroneous class predictions.

In this paper, a factor graph approach to the automated GO Annotation is presented. Briefly, a factor graph is a “bipartite graph that expresses how a global function of several variable factors into a product of local functions” [27]. Among their many applications [28, 29], factor graphs are well suited for behavioral system modeling. In this type of application, a boolean function over the variables of a system describes its valid configurations. If such a boolean function can be expressed as a set of predicates over subsets of system variables, a factor graph representation follows. Recalling that any boolean function can be represented as a rooted DAG and that domain-specific GO structures are rooted DAGs, it follows that factor graphs can also be used for GO modeling. On the other hand, factor graphs are also well-suited for the probabilistic modeling of errors arising in problems of information transmission in the presence of noise. Reminding that misclassification errors of practical binary classifiers can be formulated as an instance of such class of information theory problems, factor graphs can be also used for the probabilistic modeling of noisy GO-term predictions. Having a unique factor graph that formally includes the TPG constraint and a model of prediction noise at binary classifiers, latent GO-term predictions can be obtained from their maximum a posteriori (MAP) probability estimates. These probabilities can be in turn computed by an iterative message passing algorithm between nodes of the factor graph. To validate our proposal, the annotation of protein sequences of three biological models, *S. cerevisiae*, *A. thaliana*, and *D. melanogaster*, in the GO Molecular Function was first considered. These model organisms were chosen aiming to encompass the tree of life, representing unicellular (prokaryotic) and multicellular (plants and animals) organisms. To conclude, the annotation of four unknown proteins in *Solanum lycopersicum* with *A. thaliana* annotation data was analyzed.

## Method

We devise a method, hereafter called Factor Graph GO Annotation (FGGA) that exploits the ability of factor graphs for modeling logical and statistical constraints over system variables, e.g., the key TPG constraint over GO-term annotations or a convenient probability distribution of raw GO-term predictions over actual GO-term annotations. The FGGA approach is split into three steps. The first one deals with the construction of a core Factor Graph (FG) from a predefined GO-DAG. The second one deals with the enrichment of the core factor graph to take into account the noisy nature of GO-term predictions. Finally, the third step deals with the proper setting of a message passing algorithm to infer GO annotations of unannotated samples.

### Matching a GO-DAG to a core Factor Graph

**Fig 1:**
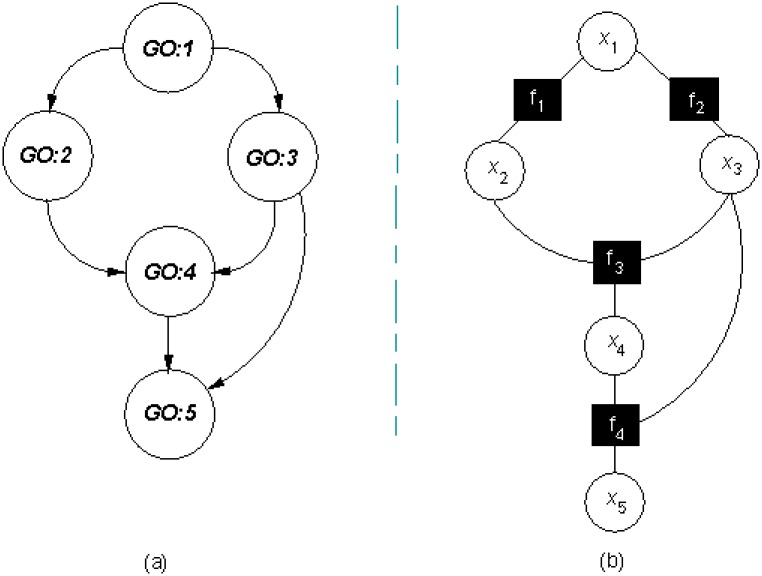
Matching a GO-DAG to a core FG. (a) GO-DAG where *GO:i* nodes are GO-terms and edges are *is*_*a* relationships (b) Core GO-FG where *x*_*i*_ are variable nodes representing positive/negative *GO:i* annotations and *f*_*k*_ are logical factor nodes modeling TPG constraint.

Given a GO subgraph, GO-terms *GO:i* are mapped to binary-valued variable nodes *x*_*i*_ of a factor graph while relationships between GO-terms are mapped to logical factor nodes *f*_*k*_ describing valid *GO:i* configurations under the TPG constraint. This matching task can be accomplished through an adapted version of the Breadth-First Search (BFS) [30] algorithm. As shown in Fig 1a, starting from the top-root node in a given GO-DAG, the identity of visited child nodes is preserved so that a new factor node between a parent and a child node is introduced only when the child node has not been previously visited; otherwise, the parent node is attached to the early created factor node for the revisited child node.

Practically, logical factor nodes *f*_*k*_ are implemented with truth tables of 2^#*child*+#*parents*^ entries. At each of these entries, the specific parent/child role of participating variable nodes is required to check the TPG constraint. As shown in Table 1 where 1/0 denotes positive/negative annotation respectively, the logical factor *f*_3_ in Fig 1b ensures that the TPG constraint over variable nodes *x*_2_, *x*_3_ and *x*_4_ is fulfilled whenever *x*_4_ is a child node and *x*_2_ and *x*_3_ are its parent nodes (multiple inheritance over *x*_4_).

**Table 1:** The truth table of the logical factor node *f*_3_. Positive/negative annotations of variable nodes *x*_2_, *x*_3_ and *x*_4_ are depicted as 1/0. Parent variable nodes *x*_2_ and *x*_3_ are shown in bold.

*x*_2_	*x*_3_	*x*_4_	*f*_3_(*x*_2_, *x*_3_, *x*_4_)
0	0	0	1
0	0	1	0
0	1	0	1
0	1	1	0
1	0	0	1
1	0	1	0
1	1	0	1
1	1	1	1

Formally, logical factor nodes *f*_*k*_ over subsets of variable nodes ensure the local satisfiability of the TPG constraint. With this aim, two logical rules are repeatedly evaluated. Specifically, if a child GO-term is annotated positive, then its parent GO-term(s) must also be annotated positive. On the other hand, if a parent GO-term is annotated negative, then its children GO-term must also be annotated negative. In predicate logic language [31], let *is*_*a*(*GO:j*, *GO:i*) denotes *GO:i* parent of *GO:j* child. Similarly, let *annotated*(⋅) denote the positive annotation of any GO-term. As a result, at least one of the following rules Eqs (1) or (2) must be active and fullfilled by any pair of GO-terms involved within a *is*_*a* relationship:

$$\begin{array}{c} r_{1}:\forall i,j\;is\_a\left(GO:j,GO:i\right)\wedge annotated\left(GO:j\right)\to annotated\left(GO:i\right) \end{array}$$ (1)

$$\begin{array}{c} r_{2}:\forall i,j\;is\_a\left(GO:j,GO:i\right)\wedge \neg annotated\left(GO:i\right)\to \neg annotated\left(GO:j\right) \end{array}$$ (2)

Regarding Table 1, let “*x*_4_ is the child of *x*_3_” and “*x*_4_ is the child of *x*_2_” denote the two *is*_*a* relations between *GO:4*, *GO:3* and *GO:2* terms. For both these relations, rule 2 is active and fulfilled at row 1 and thus, *f*_3_ is true. On the other hand, rule 1 is active for both relations at row 4 but fulfilled by only one of them and thus, *f*_3_ is false.

### Enrichment of the core GO-FG

**Fig 2:**
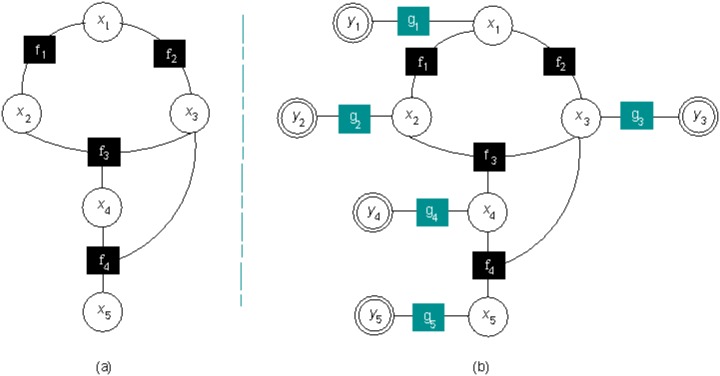
(a) Core GO-FG. (b) Enriched core GO-FG where *x*_*i*_ are latent variable nodes modeling actual positive/negative *GO:i* annotations and *f*_*k*_ are logical factor nodes modeling the TPG constraint over them, *y*_*i*_ are observable variable leaf nodes modeling real-valued *GO:i* predictions and *g*_*i*_ are probabilistic factor nodes modeling their statistical dependence on latent variable nodes *x*_*i*_.

In practice, actual *GO:i* annotations of unannotated samples are estimated as accurately as possible with binary classifiers. Therefore, variable nodes *x*_*i*_ in the core GO-FG must be considered latent behind a new class of variable leaf nodes *y*_*i*_ modeling observable, but uncertain, *GO:i* term predictions. The enrichment of the core GO-FG with variable leaf nodes *y*_*i*_ requires the introduction of a new class of probabilistic factor nodes *g*_*i*_ modeling their statistical dependence on latent variable nodes *x*_*i*_ (see Fig 2). For this purpose, a communication channel model between latent binary inputs *x*_*i*_ and observable real-valued outputs *y*_*i*_ can be assumed. Hence, latent binary inputs *x*_*i*_ (±1) corrupted with additive white zero mean Gaussian noise *z*_*i*_ of variance *η*_*i*_, so that *y*_*i*_ = *x*_*i*_+*z*_*i*_ holds, can be assumed [32]. In such a case, probabilistic factor nodes *g*_*i*_ can be set [33] to $p(x_{i}|y_{i})=\frac{1}{1+e^{\frac{-2\cdot y_{i}\cdot x_{i}}{\eta_{i}}}}$.

To completely describe a FG model for the automated GO annotation problem, the estimation of noise variances *η*_*i*_ remains to be done. Under the assumption of symmetrical conditional probability distributions for predictions *y*_*i*_ over latent annotations *x*_*i*_, variances *η*_*i*_ can be easily estimated using a validation dataset of positively/negatively annotated samples. Specifically, let *D* be a validation dataset with *L*^+^ positively annotated samples. Hence, $\hat{\eta_{i}}=\frac{1}{L^{+}-1}\sum_{l=1}^{L^{+}}{\left(y_{i}^{l}-x_{i}^{l}\right)}^{2}$ where $x_{i}^{l}=1$ is the positive annotation of the *l*-th data sample to the *GO:i* term and $y_{i}^{l}$ is the corresponding real-valued classifier prediction.

### Message passing algorithm in FGGA

Once a factor graph model *F* for the automated GO annotation problem has been defined, an iterative message passing algorithm [34] between nodes of *F* can be used to compute maximum a posteriori (MAP) estimates ${\hat{x}}_{i}$ of variable nodes *x*_*i*_ modeling actual *GO:i* annotations. Given an unannotated input sample **s**, all variable leaf nodes *y*_*i*_ in *F* gets instantiated by a set *Y*(***s***) of real-valued GO-term predictions issued by a set of base binary classifiers. Without loss of generality, let binary classifier’s outputs be characterized by a set of variances ***η*** describing conditional Gaussian probability distributions of real-valued GO-term predictions *y*_*i*_ over latent annotations *x*_*i*_. Hence, starting from instantiated variable leaf nodes *y*_*i*_, an iterative message passing algorithm is performed until some convergence criteria *ξ*, e.g. *ξ* = 10^−3^, or a maximum number *I*_*max*_ of iterations is set, e.g. *I*_*max*_ = 50. At this stage, marginal probabilities *p*(*x*_*i*_|*Y*(**s**)) can be approximated by their iterative version *p*^*t*^(*x*_*i*_|*Y*(**s**)) provided by a Sum-Product algorithm at the *t* − *th* iteration step. In message passing terms, probabilities *p*^*t*^(*x*_*i*_|*Y*(**s**)) follow from the product of the last incoming and outgoing messages at any of the *x*_*i*_ linking edges. From these probabilities, MAP estimates ${\hat{x}}_{i}$ can be computed and used to provide consistent *GO:i* annotations of unannotated samples **s** in a predefined GO domain. As a result, the following FGGA algorithm (see Alg. 1) follows:

**Algorithm 1** FGGA

**Input**:

 GO factor graph *F* with *n* GO-terms, a sample **s** to be annotated, a set *Y* of *n* predictions over **s**, a set ***η*** of prediction noise variances, a convergence criteria *ξ*, a maximum number *I*_*max*_ of iteration steps

**Output**:

 MAP estimates ${\hat{x}}_{i}$ actual GO-term annotations on **s**, *i* = 1, …, *n*

1: **for** *t* = 1 to *I*_*max*_ **do**

2:   *p*^*t*^ (*x*_*i*_ ∣ Y(**s**)) ${|}_{i=1}^{n}\leftarrow$ Sum-Product(F, ***η***, Y(**s**))

3:   **if** $|p^{t}\left(x_{i}|•\right)-p^{t-1}\left(x_{i}|•\right)|<\xi \;\forall x_{i}$ **then**

4:    break

5:   **end if**

6: **end for**

7: **return** ${\hat{x}}_{i}=\underset{x_{i}}{\mathrm{argmax}}\;p^{t}\left(x_{i}|•\right){|}_{i=1}^{n}$

## Results and Discussion

### Experimental Protocol

Three models organisms, *S. cerevisiae* [35], *A. thaliana* [36], and *D. melanogaster* [37] were considered. For each of them, *robust* and *loose* annotation datasets in the GO Molecular Function domain were generated using different subsets of GO annotation evidence codes (see Table 2). *Robust* annotation datasets were built from protein sequences with defined GO experimental evidence codes (http://geneontology.org/page/guide-go-evidence-codes), i.e., inferred from mutant phenotype (IMP), inferred from genetic interaction (IGI), inferred from physical interaction (IPI), inferred from expression pattern (IEP) and inferred from direct assay (IDA). On the other hand, *loose* annotation datasets were built from protein sequences with former experimental evidence codes, traceable author statement (TAS) evidence codes and inferred from electronic annotation (IEA) evidence codes. Recalling that a minimum amount of annotation data is required for the prediction of individual GO-terms, GO sub-graphs with a minimum of 50/10 positively annotated protein sequences per individual GO-term were respectively considered for *robust/loose* annotation datasets. To assemble conveniently balanced binary training datasets [38], positive annotated protein sequences to individual GO-terms were complemented with negative annotated instances using the *inclusive* separation policy described in [39].

**Table 2:** *S. cerevisiae*, *A. thaliana* and *D. melanogaster* datasets in the GO-Molecular Function domain.

Training	Organism	# GO-terms	Characterization	# Features	# Samples
*robust*	*S. cerevisiae*	103	Pfam	3070	3223
			Physicochemical^+^	457	3223
	*A. thaliana*	54	Pfam	3323	2863
			Physicochemical^+^	457	3856
	*D. melanogaster*	226	Pfam	4823	8636
			Physicochemical^+^	457	8636
*loose*	*S. cerevisiae*	435	Pfam	3070	3223
			Physicochemical^+^	457	3223
	*A. thaliana*	659	Pfam	3789	19601
			Physicochemical^+^	457	24150
	*D. melanogaster*	656	Pfam	4825	8655
			Physicochemical^+^	457	9320

Concerning characterization methods of individual protein sequences in terms of a fixed number of input features, the presence/absence of conserved domains in the Pfam database [40] and the measurement of 457 physicochemical/secondary structure properties, 453 of the physicochemical type [41] and four of the secondary structure type [42, 43], were considered. Pfam data was obtained for each protein as a binary vector where each element indicates the presence/absence of domains. Physicochemical and secondary structure data was obtained for each protein as a real vector where each element indicates the value of a physicochemical/secondary structure property. The choice of these baseline characterization methods was guided by the desire to develop in-silico annotation methods of broad applicability across organisms, including non-model instances for which more advanced characterizations, e.g., gene expression or protein-protein interaction data [44], are unlikely to be available. Actually, for many genes coding for proteins of unknown function and thus no significant Pfam hit, the naive physicochemical and secondary structure characterization, hereafter “Physicochemical^+^”, remains valid. Practically, protein sequence characterization methods were implemented with the help of the EMBL-EBI Pfam [45] database services and the Bio.SeqsUtils [46] package.

Concerning baseline binary classifiers, differently from [18, 19] where the costly bootstrap aggregation of hard-margin linear Support Vector Machines (SVM) classifiers with unthresholded outputs was used to fulfill the Gaussian assumption of prediction noise, single soft-margin linear SVMs with default constant complexity C = 1 were used for both the FGGA and TPR-DAG methods. To fulfill the Gaussian assumption in the FGGA approach, real valued *Y*_*i*_ predictions were set to the margin of SVM classifier outputs. On the other hand, probabilistic linear SVM outputs required by the TPR-DAG method were computed using the standard Platt’s sigmoid fitting approach. Practically, SVMs were implemented with the e-1071 R package [47].

Concerning the TPR-DAG method, the algorithm described in [25] was implemented in C++. Briefly, for each GO-term in a given GO subgraph, its maximum distance to the root node is first computed. Starting from the set of most distant GO-terms, flat SVM predictions of individual GO-terms are updated using the TPR heuristic. Therefore, a consensus prediction for each GO-term is obtained by averaging its flat SVM prediction and those of positive child GO-terms. Without loss of generality, the threshold for positive predictions is set to 0.5. This bottom-up update process over flat SVM predictions is repeated until the root node is reached. To accomplish a consistent set of hierarchical GO-term predictions, a final top-down update process on consensus GO-term predictions is performed. As a result, a child GO-term with a consensus prediction stronger than any of its parents is forced to update its value with that of its weakest parent. This process is repeated until most distant GO-terms are reached.

Concerning the evaluation of the predictive performance of the FGGA approach, a 5-fold cross-validation test was performed using the TPR-DAG algorithm as a reference comparison. To shed light on the absolute improvements of FGGA predictions, baseline SVM classifiers were also evaluated. Aiming to rank the prediction accuracy of FGGA, TPR-DAG leveraged binary classifiers and baseline SVM classifiers, per GO-term average AUC scores [48] were computed. Taking into account that GO annotation gets harder as deeper levels of the hierarchy are considered [49], prediction performance was measured by means of the hierarchical precision (HP), the hierarchical recall (HR), and the hierarchical balanced F-score (HF) reflecting their trade-off. Comparisons between the FGGA and TPR-DAG methods were performed with the Wilcoxon rank sum test at the *p*_*value*_ = 0.01 significance level. Finally, to evaluate the ability of the FGGA approach to extend biological knowledge, a molecular function annotation problem in tomato (*Solanum lycopersicum* cv. Heinz 1706) [50] was considered.

### Prediction performance on held-out data

**Fig 3:**
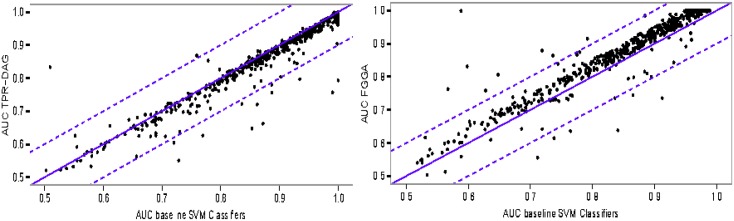
Scatter-plot of the average AUC after versus before TPR-DAG and FGGA classification. Annotation of *D. melanogaster* protein sequences to the GO-Molecular Function domain with Pfam characterization and *loose* annotation data is considered. (Left) The average AUC for TPR-DAG versus baseline SVM classifiers. (Right) The average AUC for FGGA versus baseline SVM classifiers.

Whatever the organism, characterization and training data policy, FGGA improved both baseline SVM and TPR-DAG classifiers. This was particularly evident in the annotation of *D. melanogaster* protein sequences for which the deeper, broader and richer, in terms of jumping edges, GO-DAGs were observed. As shown in Fig 3, with Pfam characterization and *loose* annotation data, both TPR-DAG and FGGA classifiers improve the average AUC of their baseline SVM classifiers. However, FGGA improvements are remarkably higher. Similar results were observed in other experimental conditions (see, e.g., S1 Fig).

**Fig 4:**
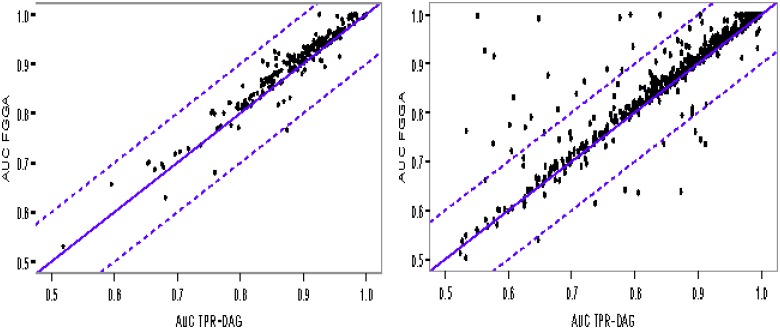
Scatter-plot of the average AUC for FGGA and TPR-DAG classifiers on the annotation of *D. melanogaster* protein sequences to the GO-Molecular Function domain with a Pfam characterization. Points above the diagonal show AUC improvements by FGGA. Points above the dashed line show 10% margin improvements. (Left) GO with 226 terms, 10 levels and *robust* annotation data. (Right) GO with 656 terms, 14 levels and *loose* annotation data.

Specific average AUC improvements of FGGA over TPR-DAG classifiers for *D. melanogaster* protein sequences and Pfam characterization are shown in Fig 4. FGGA improvements reached roughly 87% of the GO-terms, independently of the use of *robust* (198 out of 226 GO-terms) or *loose* (581 out of 656 GO-terms) annotation data at the training stage. In the latter case, a closer look revealed 21 GO-terms belonging to the deeper levels of the GO hierarchy, their minimum level was 6, with an average AUC above the 10% margin. On the other hand, only 8 GO-terms above the 10% margin where identified for TPR-DAG classifiers. Conversely, these GO-terms belonged to rather superficial levels, their maximum level was 5, of the GO hierarchy. Similar results were obtained for *A. thaliana* (see S1 Fig), *S. cerevisae* (see S2 Fig) and for the Physicochemical^+^ characterizacion (see S3 Fig). Overall, these results suggest the usefulness of the FGGA approach for tackling specific GO annotations in the presence of limited amounts of annotation data.

Average AUC results per individual GO-term correlate well with hierarchical metrics of annotation performance. As shown in Table 3, whatever the experimental arrangement, FGGA outperforms TPR-DAG based on hierarchical F-score results (*p*_*value*_ = 0.01). This is consistent with hierarchical precision and recall results showing FGGA improvements over TPR-DAG in at least 8 of 12 experimental instances and equaling in remaining cases. Furthermore, although the use Physicochemical^+^ characterization strongly reduces hierarchical precision levels in both methods, a more precise annotation performance is still accomplished by FGGA which outperforms TPR-DAG in 5 of the 6 experimental instances (*p*_*value*_ = 0.01). For the sake of completeness, hierarchical F-scores of baseline SVM classifiers were also evaluated. As expected, the value of consistent FGGA predictions against independent ones in baseline SVM classifiers can be clearly appreciated (see S1 Table).

**Table 3:** Average hierarchical precision(HP), recall (HR) and F-score (HF) of the FGGA and TPR-DAG methods in the GO Molecular Function. Organisms are *S. cerevisiae*, *A. thaliana* and *D. melanogaster*. Characterizations are Pfam and physicochemical/secondary structure (PhyChe^+^) properties. Training policies are *robust* and *loose*. For each model organism, characterization and training policy, the best performing method according to the Wilcoxon rank sum test (*p*_*value*_ = 0.01) is shown in bold.

Organism	Characterization	Training	Method	HP	HR	HF
*S. cerevisiae*	Pfam	*robust*	FGGA	0.62	**0.76**	**0.64**
			TPR-DAG	0.62	0.66	0.61
		*loose*	FGGA	0.53	**0.78**	**0.60**
			TPR-DAG	0.53	0.70	0.56
	PhyChe^+^	*robust*	FGGA	0.46	**0.81**	**0.57**
			TPR-DAG	0.45	0.79	0.55
		*loose*	FGGA	**0.46**	0.84	**0.54**
			TPR-DAG	0.40	0.83	0.52
*A. thaliana*	Pfam	*robust*	FGGA	**0.74**	**0.80**	**0.70**
			TPR-DAG	0.71	0.73	0.69
		*loose*	FGGA	**0.78**	0.90	**0.80**
			TPR-DAG	0.76	0.90	0.77
	PhyChe^+^	*robust*	FGGA	**0.49**	**0.86**	**0.60**
			TPR-DAG	0.47	0.84	0.59
		*loose*	FGGA	**0.37**	**0.87**	**0.50**
			TPR-DAG	0.33	0.84	0.46
*D. melanogaster*	Pfam	*robust*	FGGA	0.71	**0.86**	**0.75**
			TPR-DAG	0.70	0.81	0.72
		*loose*	FGGA	**0.57**	**0.82**	**0.65**
			TPR-DAG	0.51	0.80	0.59
	PhyChe^+^	*robust*	FGGA	**0.43**	0.84	**0.55**
			TPR-DAG	0.40	0.84	0.52
		*loose*	FGGA	**0.37**	**0.87**	**0.50**
			TPR-DAG	0.33	0.85	0.47

Complementary evaluations were performed to shed some light on the relation between the variance of base binary classifiers and FGGA hierarchical F-scores when increasing annotation noise. This would be the case of using the naive Physicochemical^+^ characterization or the *loose* training policy. As expected, whatever the organism, the variance of base binary classifiers augmented with the Physicochemical^+^ characterization and these variations were more evident with the *loose* training policy. For *D. melanogaster* and robust training, variances of base binary classifiers grew from an average of 0.27 to 0.33 when changing from the Pfam to the Physicochemical^+^ alternative. This was consistent with a reduction of the hierarchical F-score from 0.75 to 0.55. Likewise, with loose training, variances grew from an average of 0.30 to 0.41 with a reduction of the F-scores from 0.65 to 0.50. Similar results were observed for the other two model organisms. Overall, these results suggest that within the FGGA framework, augmenting the confidence of binary base classifiers by reducing their variances may be effectively rewarded by improving annotation performance.

### Annotation of proteins of unknown function in plants

The physical distribution of gene in plants seems to be not random and physical clusters of genes with related functional classes can be expected [51]. We consider a GO Molecular Function annotation problem in *Solanum lycopersicum* cv Heinz 1706. Four small heat shock protein (*shsp*) genes [52], Solyc06g076520, Solyc06g076540, Solyc06g076560 and Solyc06g076570, of well-known chaperone function in fruit ripening and heat shock stress [53, 54] map together to a ∼ 15 Kbp region in chromosome 6 suggesting the existence of a region of functional related genes. In a wider region of ∼ 30 Kbp, these genes map together with a Phosphoserine phosphatase SerB gene (Solyc06g076510) and four genes of unknown function, Solyc06g076500, Solyc06g076530, Solyc06g076580 and Solyc06g076590.

We hypothesize that FGGA classifiers trained on *loose* *A. thaliana* annotation datasets characterized by naive physicochemical/secondary structure properties may shed some light on the four genes of unknown function in *Solanum lycopersicum*. To support this hypothesis, Solyc06g076540 and Solyc06g076510 were first used as positive controls. Since Solyc06g076540 lacks of a GO molecular function annotation, the “protein-self association” annotation of its HSP17.8 ortholog in *A. thaliana* was used. On the other hand, “phosphatase activity” and “magnesium ion binding” GO annotations were used for Solyc06g076510. Recalling that for hierarchical classifiers, a prediction is considered correct provided it is included in the predicted graph, the two controls were satisfied (see S5 and S6 Figs). Based on these positive annotation results, FGGA predictions on the four genes of unknown function were performed.

Aiming to recover most specific and confident FGGA predictions for guiding experimental studies, a cut threshold of 0.95 for leaf nodes was set for the analysis predicted graphs, i.e., leaf GO-terms whose estimated probabilities were below the threshold were disregarded. For Solyc06g076500, a subgraph containing 122 out of the 659 original GO-terms, 54 of them being leaf GO-terms, was recovered (see S7 Fig). Among the top five scoring leaf GO-terms, GO:0016893 -endonuclease activity, active with either ribo- or deoxyribonucleic acids- whose ancestor is GO:0004518 -nuclease activity- appears as a candidate annotation term [55]. For Solyc06g076530, a subgraph containing 185 out of the 659 original GO-terms, 75 of them being leaf GO-terms, was recovered. Among the top five scoring leaf GO-terms, GO:0004722 -protein serine/threonine phosphatase activity- whose ancestor is GO:0016791 -phosphatase activity- appears as a candidate annotation term [56]. For Solyc06g076580, a subgraph containing 52 out of the 659 original GO-terms, 21 of them being leaf GO-terms, was recovered. Among the top five scoring leaf GO-terms, GO:0016209 -antioxidant activity- appears as a candidate annotation term [57]. Finally, for Solyc06g076590, a subgraph containing 45 out of the 659 original GO-terms, 31 of them being leaf GO-terms, was recovered. Among the top five scoring leaf GO-terms, GO:0046983 -protein dimerization activity- whose ancestor is GO:0005515 -protein binding- appears as a candidate annotation term [58]. Altogether, these results suggest that all genes in the target region could be involved in a chaperone network induced during fruit ripening or heat shock stress [59, 60].

## Conclusions

A factor graph based method for automated GO annotation has been presented. The method, called FGGA, embodies elements of predicate logic, communication theory, supervised learning and inference in graphical models. Elements of predicate logic allow a formal, yet intuitive, treatment of relationships between GO-terms. Although only the main *is*_*a* relationship has been practically considered, other types of transitive relationships, such as *part of* or *has part*, are also possible. Likewise, elements of communication theory allow a formal, yet practical, treatment of the uncertainty in practical GO-term predictions. Since these predictions are issued by practical binary classifiers, key factors affecting the generalization performance of the resulting ensemble can then be practically considered. In particular, under the assumption of a Gaussian communication channel model corrupting ideal GO-term predictions, the variances of base binary classifiers can be used to model their individual confidences. Similarly, under the assumption of linear soft-margin SVM binary classifiers, observed margins can be used to model the confidence of GO-term predictions. Both types of confidences, known to affect the generalization of overall ensemble classifiers [61, 62], are fully exploited within the FGGA framework. This is accomplished at the FGGA inference stage with an adapted version of the widely used sum-product algorithm of factor graphs. This iterative message passing algorithm is used to approximate MAP of latent GO-term annotations. Evaluations on *S. cerevisiae*, *A. thaliana*, and *D. melanogaster* protein sequences suggest that improvement of the correctness (precision) and the completeness (recall) of annotation results with respect to the TPR-DAG heuristic is the payoff for FGGA modeling efforts. In this regard, an insight into the power of the FGGA approach for studying proteins of unknown function has been presented.

Although throughout this paper only the automated annotation of protein sequences has been practically considered, the annotation of other types of striking functional gene products is also possible, e.g., long non-coding RNAs [63]. Since these RNA sequences are weakly conserved across species [64] except in mammals [65], uncovering their functional annotation entails a challenging bioinformatics problem [66]. FGGA may bring an opportunity for improving the annotation of long non-coding RNA sequences through boosting the confidence of base binary classifiers by the integration of multiple sources of annotation data, e.g., Rfam database [67]. Interestingly, the complexity of such integration process could remain hidden at base binary classifiers.

## Supporting Information

S1 Fig TPR-DAG and FGGA versus baseline SVM classifiers. Scatter-plot of the average AUC after versus before TPR-DAG and FGGA classification. Annotation of *A. thaliana* protein sequences to the GO-Molecular Function domain with Physicochemical^+^ characterization and *loose* annotation data is considered. (Left) The average AUC for TPR-DAG versus baseline SVM classifiers. (Right) The average AUC for FGGA versus baseline SVM classifiers.
(EPS)

S2 Fig FGGA versus TPR-DAG on *A. thaliana* with Pfam characterization. Scatter-plot of the average AUC for FGGA and TPR-DAG classifiers on the annotation of *A. thaliana* protein sequences to the GO-Molecular Function domain with a Pfam characterization. Points above the diagonal show AUC improvements by FGGA. Points above the dashed line show 10% margin improvements. (Left) GO with 54 terms, 6 levels and *robust* annotation data. (Right) GO with 659 terms, 14 levels and *loose* annotation data.
(EPS)

S3 Fig FGGA versus TPR-DAG on *S. cerevisae* with Pfam characterization. Scatter-plot of the average AUC for FGGA and TPR-DAG classifiers on the annotation of *S. cerevisae* protein sequences to the GO-Molecular Function domain with a Pfam characterization. Points above the diagonal show AUC improvements by FGGA. Points above the dashed line show 10% margin improvements. (Left) GO with 103 terms, 10 levels and *robust* annotation data. (Right) GO with 435 terms, 14 levels and *loose* annotation data.
(EPS)

S4 Fig FGGA versus TPR-DAG on *A. thaliana* with Physicochemical^+^ characterization. Scatter-plot of the average AUC for FGGA and TPR-DAG classifiers on the annotation of *A. thaliana* protein sequences to the GO-Molecular Function domain with a Physicochemical^+^ characterization. Points above the diagonal show AUC improvements by FGGA. Points above the dashed line show 10% margin improvements. (Left) GO with 54 terms, 6 levels and *robust* annotation data. (Right) GO with 659 terms, 14 levels and *loose* annotation data.
(EPS)

S5 Fig Positive control Solyc06g076540. Predicted graph for Solyc06g076540 using *A. thaliana* *loose* annotation data for FGAA training and a Physicochemical^+^ characterization. The graph contains 171 GO-terms. Annotation control is shown in white.
(EPS)

S6 Fig Positive control Solyc06g076510. Predicted graph for Solyc06g076510 using *A. thaliana* *loose* annotation data for FGGA training and a Physicochemical^+^ characterization. The graph contains 162 GO-terms. Annotation control is shown in white.
(EPS)

S7 Fig Solyc06g076500 of unknown molecular function. Prunned graph containing 122 GO-terms, 54 of them being leaf nodes, for the prediction of Solyc06g076500 molecular function. *A. thaliana* *loose* annotation data with a Physicochemical^+^ characterization is used for FGGA training. Leaf prunning with a cut threshold of 0.95 is considered. Top five scoring leaf GO-terms along with their MAP estimates are shown in blue.
(EPS)

S1 Table Table FGGA and baseline SVM classifiers. Average hierarchical precision(HP), recall (HR) and F-score (HF) of the FGGA method and baseline SVM classifiers (Flat) in the GO Molecular Function. Organisms are *S. cerevisiae*, *A. thaliana* and *D. melanogaster*. Characterizations are Pfam and physicochemical/secondary structure (PhyChe^+^) properties. Training policies are *robust* and *loose*. For each model organism, characterization and training policy, the best performing method according to the Wilcoxon rank sum test (*p*_*value*_ = 0.01) is shown in bold.
(EPS)
